# Mechanistic and Kinetic Analysis of Perovskite Memristors with Buffer Layers: The Case of a Two-Step Set Process

**DOI:** 10.1021/acs.jpclett.2c03669

**Published:** 2023-02-04

**Authors:** Cedric Gonzales, Antonio Guerrero

**Affiliations:** Institute of Advanced Materials (INAM), Universitat Jaume I, 12006 Castelló, Spain

## Abstract

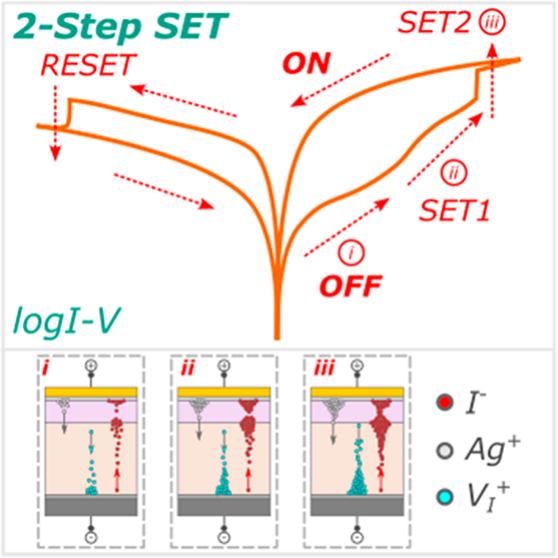

With the increasing demand for artificially intelligent hardware systems for brain-inspired in-memory and neuromorphic computing, understanding the underlying mechanisms in the resistive switching of memristor devices is of paramount importance. Here, we demonstrate a two-step resistive switching set process involving a complex interplay among mobile halide ions/vacancies (I^–^/V_I_^+^) and silver ions (Ag^+^) in perovskite-based memristors with thin undoped buffer layers. The resistive switching involves an initial gradual increase in current associated with a drift-related halide migration within the perovskite bulk layer followed by an abrupt resistive switching associated with diffusion of mobile Ag^+^ conductive filamentary formation. Furthermore, we develop a dynamical model that explains the characteristic *I*–*V* curve that helps to untangle and quantify the switching regimes consistent with the experimental memristive response. This further insight into the two-step set process provides another degree of freedom in device design for versatile applications with varying levels of complexity.

Artificially intelligent devices have recently been attracting considerable attention due to the increasing hardware demands of neural network configurations with varying levels of computational complexity.^1−3^ Resistive random-access memory (ReRAM) based on memristor devices is widely considered as the most promising candidate for next-generation computational frameworks owing to their in-memory and neuromorphic computing capabilities.^3−5^ This includes their simple device structure, high device density, low power consumption, fast switching speed, and monolithic integration compatibility with existing complementary metal/oxide/semiconductor (CMOS) systems.^4,6,7^ Resistive switching has been demonstrated in a diverse range of devices such as metal/oxide/metal structures,^8−10^ organic semiconductors,^11−13^ CMOS-compatible silicon-based devices,^14−16^ and numerous halide perovskite formulations.^17−19^

Different neuromorphic computing schemes require specific switching properties from artificial neuromorphic hardware. These characteristics range from nonvolatile binary switching for digital in-memory computing and spiking neural networks^1,3^ to volatile analog switching for artificial neural network configurations and brain-inspired computing.^4,20−22^ The device application is intimately associated with the switching mechanism. In this respect, proposed resistive switching mechanisms range from drift-related nonfilamentary oxygen vacancy migration and redistribution in metal and titanium oxide-based devices^23,24^ and diffusive formation and rupture of metallic conductive filaments in silicon oxide-based devices^15,16^ to halide filamentary formation followed by electrochemical interactions with contacts that promote switching in perovskite-based devices.^25,26^ Despite the rapid development of material systems and configurations exhibiting distinct memristive switching properties, understanding the precise mechanisms is essential for tailoring device design for a wider range of applications in more advanced computational frameworks.

Metal halide perovskite materials are versatile candidates for memory applications as they benefit from mixed ionic–electronic conduction due to ionic halide defect displacement resulting in intrinsic memory effects.^27−29^ Metal halide perovskites have a chemical structure of ABX_3_, where A is a monovalent cation (i.e., MA = CH_3_NH_3_^+^), B is a divalent cation (i.e., Pb^2+^), and X is a halide anion (i.e., I^–^). With the flexibility of the perovskite structure as the material platform, it possesses a broad range of switching physics suitable for a wide variety of neuromorphic computing achitectures.^30^ Perovskite-based memristive devices have been demonstrated to function as artificial synapses exhibiting essential synaptic behaviors for neuromuscular systems, pupil reflex, and light-sensitive optogenetic applications.^31−34^ Additionally, integration of two-dimensional structures,^35,36^ mixed formulations,^37,38^ and nanocrystals^21,39^ further increases the already vast degrees of freedom or state variables in perovskite-based memristors allowing tunability and versatility specific to the desired implementation. However, with the inclusion of various intermediate buffers,^40,41^ a complete picture of the resistive switching mechanism is imperative for tailoring the design of reliable memristive devices for more versatile applications.

Here, we present a two-step resistive switching (RS) SET process in methylammonium lead iodide (MAPbI_3_) memristors with various thin undoped buffer layers. The MAPbI_3_ perovskite formulation is selected to gain a simple and well-established understanding of the electronic and ionic dynamics with a low activation energy. The two different buffer layers used are (6,6)-phenyl C61 butyric acid methyl ester (PCBM), typically used as an electron selective layer,^42^ and the insulator poly(methyl methacrylate) (PMMA), typically used as a protective layer for the perovskite.^43−45^ On the basis of the switching characteristics of the memristor devices, in conjuction with a direct comparison to a device configuration without a buffer layer, two distinct switching regimes are untangled: (1) an initial gradual increase in current associated with drift-related I^–^ ion and V_I_^+^ defect migration and redistribution within the perovskite bulk layer followed by (2) an abrupt resistive switching associated with diffusion-related Ag^+^ filamentary formation irrespective of the buffer layer. The two-step SET process exhibits both drift and diffusive mechanisms, depending on the applied field, allowing memristor device designs specifically tailored for targeted neural network configurations.

The cross-sectional scanning electron microscopy (SEM) micrographs of the fabricated memristor devices with PCBM and PMMA layers are shown in panels a and b of Figure 1, respectively, with the device configuration indicating the layers. Both devices have MAPbI_3_ perovskite layers with comparable thicknesses (∼400 nm) and similar crystal morphologies. The buffer layers are substantially thin with thicknesses ranging from ∼5 to ∼10 nm to have a minimal voltage drop within these layers. The characteristic *I*–*V* curves of a representative memristor device with the PCBM buffer layer is shown in Figure 1c, and RS activation and deactivation involve migration of I^–^/V_I_^+^ and Ag^+^. The memristive response requires initial conditioning of the fresh device (electroforming process) at relatively high voltages, which produces a significant change in the device conductance.^46−50^ From the fresh state of the device, a positive voltage sweep is applied to promote the electroforming process (Figure 1d). As the applied voltage approaches ∼0.9 V, the current gradually increases and the continued voltage sweep abruptly increases the device current at ∼1.45 V. A cutoff current of 50 mA is imposed on the measurement to avoid the irreversible processes that can lead to complete device breakdown. This cutoff current is reached at ∼1.6 V, where the scan direction is immediately reversed back to 0 V. Another scan toward positive bias reveals a new stabilized high-resistance state (HRS). A positive voltage sweep gradually increases in current at a first threshold voltage of *V*_Th1_ ∼ 0.25 V [SET1 process (Table 1)] and then abruptly switches ON to the low-resistance state (LRS) at a second threshold voltage of *V*_Th2_ ∼ 0.56 V (SET2 process) with an ON/OFF ratio of ∼21.3. This two-step SET process indicates two distinct switching regimes in the different voltage ranges as illustrated in Figure 1d, which will be discussed in detail below. The memristor device, then, stays at the LRS on the reverse scan direction and finally switches OFF to the HRS (RESET process) at *V*_RESET_ ∼ −0.59 V. Once electroforming has taken place, the *I*–*V* curve is stabilized and the curves of multiple cycles overlay as shown in Figure S1. Both devices exhibit ON state retention times approcaching 10^5^ s at a read voltage (*V*_read_) of 0.2 V with an endurance of >50 cycles (Figure S2), and we note some performance degradation is observed during the initial 50 cycles. It is worth noting that the memristor device configuration is designed to emphasize the kinetics and dynamics of the resistive switching mechanism. Incorporation of a large cation dopant, such as ethylenediammonium (en), has been proven to substatially improve the device stability to record endurances of 1.2 × 10^4^ cycles.^30^

**Figure 1:**
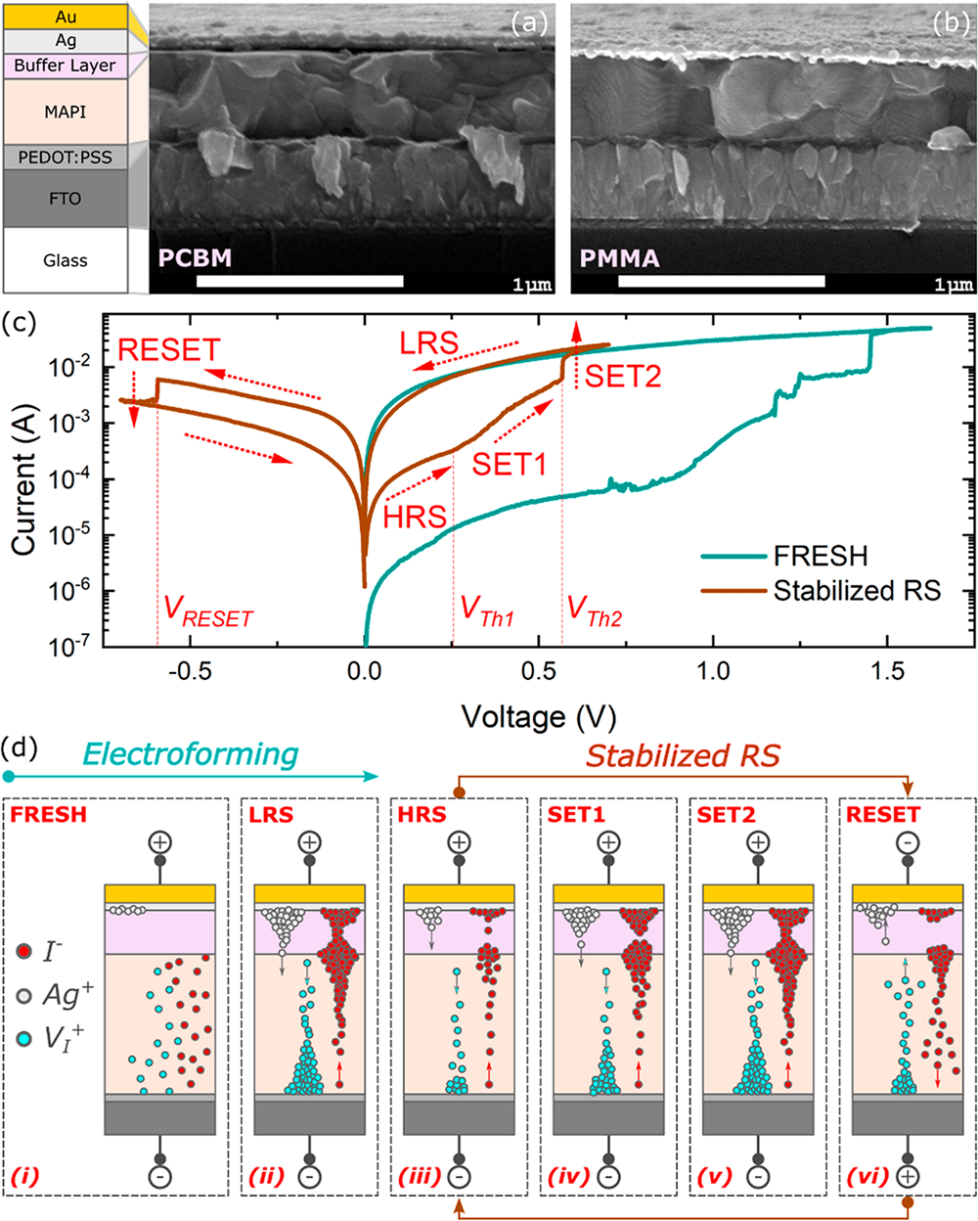
Cross-sectional SEM micrographs of memristors with thin undoped (a) PCBM and (b) PMMA buffer layers with a schematic diagram of the device configuration. (c) Representative characteristic *I*–*V* curves of the initial conditioning step of the FRESH device and the stabilized two-step SET process and RESET process with the arrows indicating the scan direction. (d) Schematic diagram of the electroforming and two-step SET process mechanism under the applied voltage of the memristive devices with buffer layers. The colors of the device configuration indicate the layers in the schematic diagram.

**Table 1:** Summary of Threshold Voltages *V*_Th1_, *V*_Th2_, and *V*_RESET_ Corresponding to the SET1, SET2, and RESET Processes, Respectively, and ON/OFF Ratios for All Memristor Devices

device	*V*_Th1_ (V)	*V*_Th2_ (V)	*V*_RESET_ (V)	ON/OFF ratio
PCBM	+0.25	+0.56	–0.59	21.3
PMMA	+0.26	+0.60	–0.59	39.3
MAPbI_3_/Au	+0.57	–	+0.29	21.0

To investigate the effect of the buffer layer on the switching properties, the performance of memristors containing either PCBM or PMMA as the buffer layer is compared with that of devices that contain no buffer layer. PCBM and PMMA are materials very different from the point of view of their electronic properties, which will help to rule out any effects of energy level aligments that affect charge extraction. Indeed, while PCBM is a semiconductor that is often used a an electron selective layer, PMMA is an insulating material. The representative stabilized responses of their memristors are shown in panels a and b of Figure 2, respectively. Both memristors feature a resistive switching with the two-step SET process as described above with similar threshold values and current levels (Table 1). Therefore, effects related to energy level alignemnt at the perovskite interfaces do not appear to be related to the switching mechanims, and the resistive switching mechanism is of a different nature as explained below. The resistive switching with the SET and RESET processes occurring at opposite polarities of the applied voltage indicates a nonvolatile bipolar resistive switching characteristic of the memristor device.^40,45,51^ Alternatively, Figure 2c shows the characteristic *I*–*V* response of a memristor without any buffer layer and a nonreactive contact (MAPbI_3_/Au). Interestingly, this device exhibits only the gradual SET1 process but without the abrupt SET2 process at the positive polarity. In addition, a gradual RESET process to the HRS in the reverse scan direction is observed. The SET and RESET processes occur at the same polarity, indicating that the device works as a volatile unipolar resistive switch.^21,51^ Therefore, a volatile memory device is transformed into a nonvolatile device by adding a buffer layer, indicating that the buffer layer acts not only as a physical barrier to ions but also as a “pool” of ions that become trapped when the external electrical field is removed.

**Figure 2:**
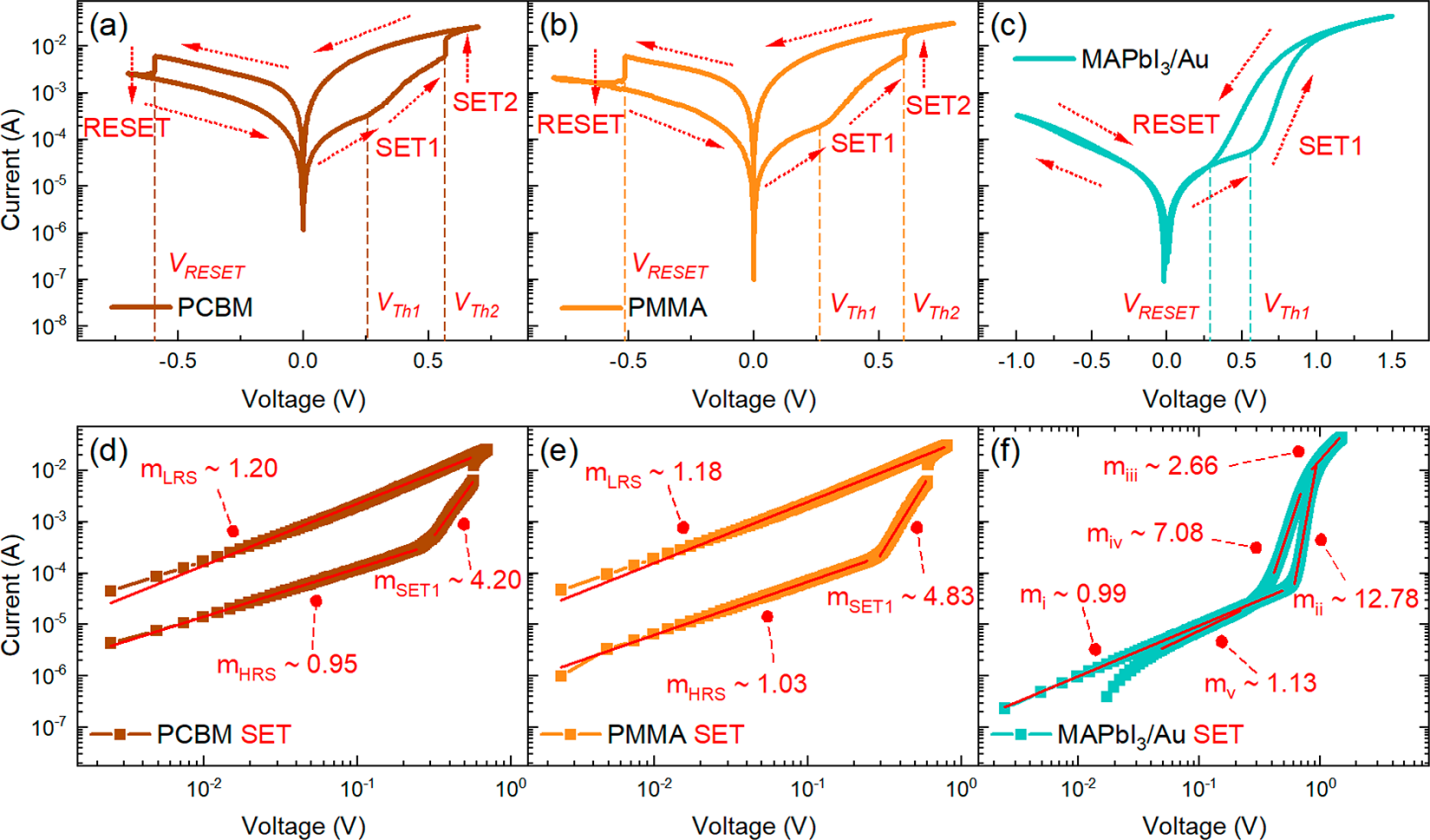
Characteristic *I*–*V* curves on the semilog scale of the memristor devices with thin undoped (a) PCBM and (b) PMMA buffer layers exhibiting nonvolatile bipolar resistive switching and (c) the MAPbI_3_/Au device exhibiting volatile unipolar resistive switching. The arrows indicate the scan direction and the corresponding SET and RESET processes with their respective threshold voltages. Corresponding SET processes on the log–log scale of the (d) PCBM, (e) PMMA, and (f) MAPbI_3_/Au devices with the slopes calculated via piecewise linear fitting.

With regard to the activation mechanims, different authors have established that for devices that do not contain a buffer layer in the presence of a nonreactive contact (i.e., Au), the gradual increase in current is attributed to the mobile I^–^ ions migrating toward the Ag contact, consequently doping the intermediate buffer layer (Figure 1d).^26,27,52,53^ Correspondingly, the iodine vacancies (V_I_^+^) migrate toward the inert PEDOT:PSS, leaving behind a doped perovskite material.^25,54^ In addition, the chemical interactions of migrating ions with the external contact can also lead to a reduction of the extraction barriers. Note that if the external contact is a reactive metal (i.e., Ag), a fast electrochemical reaction masks the memristive response as shown for the MAPbI_3_/Ag/Au interfaces in Figure S3. Alternatively, when buffer layers are used, we propose that the electroforming process at high applied voltages pushes the I^–^ ions to cross the nonconductive buffer layer, accumulating at the Ag contact leading to the formation of AgI (Figure 1d).^25,26^ The electrochemical reaction generates mobile Ag^+^ ions that migrate toward the bottom contact under the influence of the applied field. Note that neutral Ag does not have a driving force to follow the electrical field. The migration and accumulation of mobile Ag^+^, I^–^, and V_I_^+^ eventually switch the device abruptly to the LRS or ON state. Once the applied voltage returns to 0 V, the mobile Ag^+^, I^–^, and V_I_^+^ relax and a new stabilized HRS or OFF state is established. In addition, the thin Ag/Au contact is used to control and modulate the interactivity of the mobile I^+^ ions with Ag to prevent the formation of an excessively thick AgI structure with a low ionic conductivity.^55^ In general, the presence of the buffer layer in conjuction with the reactive Ag contact is responsible for the abrupt SET2 process by controlling the formation of a pool of slow-moving ions within the buffer layer and the perovskite and by regulating the reactivity of migrating ions with Ag.

We next set out to further analyze the characteristic *I*–*V* response to understand if the kinetics of the SET processes can be correlated between different samples, providing further insight into the mechanisms governing the switching processes. The SET processes from the HRS to the LRS for all memristor devices are represented in the log–log scale as shown in Figure 2b. At applied voltages below *V*_Th1_, the memristor devices are in the HRS with the current exhibiting a linear dependence on the applied voltage. Correspondingly, the calculated HRS slopes via piecewise linear fitting result in *m*_HRS_ values of ∼1 for all memristor devices, indicating an ohmic conduction mechanism.^45,49,56^ Beyond *V*_Th1_, the current gradually increases (SET1) with *m*_SET1_ values of ∼4.20 and ∼4.83 for the memristors with PCBM and PMMA buffer layers, respectively. *m*_SET1_ values of >1 are attributed to a drift-related switching mechanism due to halide ion migration and redistribution within the perovskite material.^57,58^ Once the applied voltage reaches *V*_Th2_, the memristor devices with buffer layers abruptly switch (SET2) to the LRS, which is indicative of diffusion-related conductive filament formation across the buffer layer.^22,59−61^ In the reverse scan direction, the samples with the buffer layers maintain an ohmic conduction response during the reverse scan with an *m*_LRS_ of ∼1. On the contrary, the MAPbI_3_/Au device exhibits an ohmic HRS with a slope of *m*_i_ ∼ 0.98 for applied voltages below *V*_Th1_. As the applied voltage is further increased, the SET1 process exhibits a higher *m*_ii_ of ∼12.78. This higher *m*_ii_ of MAPbI_3_/Au could be attributed the reduced series resistance of the devices, as compared to those with buffer layers. Thus, at a given applied external voltage, the electrical field present at the perovskite layer is higher than that of the devices with the buffer layers, promoting a fast migration of ions and more current injection. Once switched ON, the device then stays in the LRS with an *m*_iii_ of ∼2.66 in the reverse scan direction followed by a gradual decrease in current with an *m*_iv_ of ∼7.08 and eventually switching the device OFF back to the ohmic HRS with an *m*_v_ of ∼1.13.

As the characteristic *I*–*V* response of the memristor devices is carried out under dark and controlled conditions inside a glovebox, the measurement protocol is identical to that of the space-charge-limited conduction (SCLC) characterization.^22,45,49,62^ In SCLC analysis, the conduction mechanism at different voltage ranges is interpreted from the obtained slopes via piecewise linear fitting. However, the SCLC characterization considers only the carrier transport and injection properties of the device. In lieu of the SCLC analysis, we present a dynamical model for elucidating the experimental observations and provide a clearer understanding of the complex features of the characteristic *I*–*V* resistive switching response of the memristor devices with buffer layers. Our model takes into account interfacial reactions and charge accumulation and is not solely based on carrier transport mechanisms. This general model lends an alternative analysis pathway to various approaches such as SCLC,^22,62^ drift–diffusion simulations,^16,63^ and SPICE modeling.^64−67^

The dynamical model consists of a system of equations describing the characteristic *I*–*V* response given by^68,69^
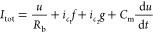
1
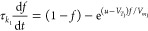
2
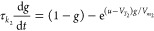
3

The model considers four contributions for the extracted current (*I*_tot_) under the applied electrical field. *u*/*R*_b_ is the ohmic conduction response term with a constant resistance *R*_b_. *i*_*c*_1__*f* is the gradual injection current term with a saturation value of *i*_*c*_1__ controlled by an occupation function *f* (0 ≤ *f* ≤ 1) associated with the SET1 process. *i*_*c*_2__*g* is the subsequent current transition term with a saturation value of *i*_*c*_2__ controlled by a different occupation function *g* (0 ≤ *g* ≤ 1) associated with the SET2 process, and the capacitive charging of the interfaces term with a capacitance *C*_m_.^68−70^ The four independent variables contribute to the total current *I*_tot_, the applied voltage (*u*), and occupation functions *f* and *g*. Both eqs 2 and 3 represent the diffusion or migration time of ions introducing a delayed response that lags behind the applied voltage with characteristic times τ_*k*_1__ and τ_*k*_2__, respectively. The delay equations are controlled by onset potentials *V*_*T*_1__ and *V*_*T*_2__, and ideality factors  and  for eqs 2 and 3, respectively, where *q* is the electron charge, *k*_b_ is the Boltzmann constant, and *T* is room temperature. When the time derivative is suppressed, the steady state solutions become
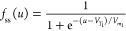
4
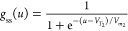
5Hence, the steady state current is then expressed as
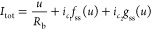
6

Applying the dynamical model (eq 6) to the experimental results provides valuable information about the resistive switching of the memristor devices with buffer layers. The fitted SET process characteristic *I*–*V* responses of the memristor devices on the linear, semilog, and log–log scales are presented in Figure 3. The detailed fitting method is described in the Supporting Information to determine the relevant parameters as summarized in Table 2. The fitted curves capture the pertinent features of ohmic LRS and the two-step SET process of the memristor devices from the linear to the log–log scales. From the extracted parameters of the fitting, the memristor devices have different *R*_b_ parameters. The *R*_b_ values can be attributed to the differences in buffer layer intrinsic resistivity and slight variations in layer thicknesses. In addition, SET1 onset potential *V*_1_ and saturation current *i*_*c*_1__ of the PCBM and PMMA devices have similar values. Moreover, both devices have comparable *m*_1_ ideality factor values of ∼2.6 and ∼3.3 for the memristor with PCBM and PMMA buffer layers, respectively. These ideality factor values can be attributed to the slow and gradual migration and reaction of ions^18,70^ or the decrease in the surface barrier at the perovskite/contact interface.^70−72^ Similarly, SET2 onset potential *V*_2_ and saturation current *i*_*c*_2__ of the PCBM and PMMA devices also have similar values. Notably, ideality factors *m*_2_ of the memristor devices are significantly lower than their corresponding *m*_1_ values with values of ∼0.01 and ∼0.03 for the devices with PCBM and PMMA buffer layers, respectively. The lower *m*_2_ values capture the abrupt resistive switching and the continued current increase after the SET process, suggesting that the conductive filamentary formation is an avalanche effect. The high correlation of the fitted SET1 parameters indicates that the mechanism during the gradual current increase is general in both devices regardless of the type of buffer layer. On the contrary, the differences in the *m*_2_ parameters suggest that the avalanche SET2 process is affected by the slight variations in the intrinsic properties and layer thicknesses of the buffer layers.

**Figure 3:**
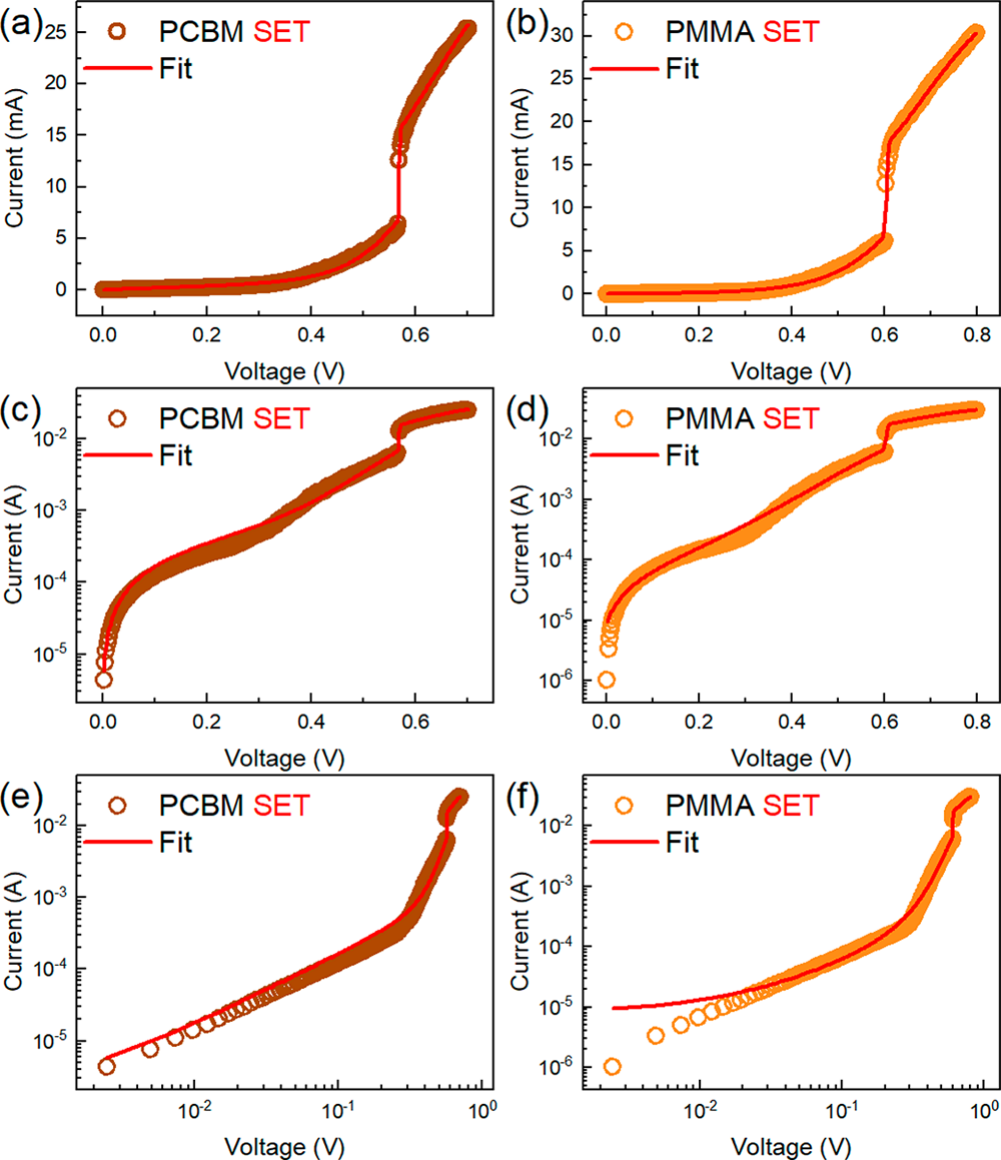
The characteristic *I*–*V* SET process with the corresponding fitted curves using the dynamical model for both memristor devices in the (a and b) linear, (c and d) semilog, and (e and f) log–log scales.

**Table 2:** Summary of the Fitted Parameters with the Corresponding Ideality Factors *m* Given by the Equation *V*_*m*_ = *mk*_b_*T*/*q* Using the Dynamical Model for All Memristor Devices

device	*R*_b_ (Ω)	*V*_*T*_1__ (V)	*V*_*m*_1__ (V)	*m*_1_	*i*_*c*_1__ (A)	*V*_*T*_2__ (V)	*V*_*m*_2__ (V)	*m*_2_	*i*_*c*_2__ (A)
PCBM	6.39 × 10^2^	6.36 × 10^–1^	6.81 × 10^–2^	2.619	2.22 × 10^–2^	5.69 × 10^–1^	5.77 × 10^–4^	0.010	8.69 × 10^–3^
PMMA	2.77 × 10^3^	6.92 × 10^–1^	8.65 × 10^–2^	3.327	2.53 × 10^–2^	6.04 × 10^–1^	1.89 × 10^–3^	0.032	1.05 × 10^–2^

On the basis of these results, a two-step resistive switching SET process is proposed involving the complex interplay among mobile Ag^+^, I^–^ ,and V_I_^+^ ions as schematically presented in Figure 1d. In the initial device state (FRESH state), (i) the mobile I^–^ ions and V_I_^+^ defects are uniformly distributed throughout the perovskite bulk layer. During the electroforming process, migrating I^–^ ions and V_I_^+^ defects accumulate at the top and bottom contacts, respectively, (ii) switching the device to the LRS. The implemented cutoff current prevents device breakdown and the irreversible conductive filamentary formation that can permanently keep the device in the LRS state. The immediate reverse scan direction back to 0 V relaxes the ion and defect migration, resulting in the rupture of conductive filaments and (iii) maintaining a stabilized HRS lower than that of the equilibrium FRESH state. From the new lower stabilized HRS with an ohmic resistance *R*_b_, a positive scan allows mobile I^–^ ions to migrate toward the Ag contact, consequently doping the intermediate buffer layer.^26,52^ Correspondingly, the V_I_^+^ defects migrate toward the inert PEDOT:PSS and FTO bottom contacts.^25,54^ As the device is already at a less resistive HRS, a lower applied voltage (*V*_Th1_) is required for the migrating I^–^ ions and V_I_^+^ defects to accumulate at the contacts, (iv) gradually switching the resistance state of the device (SET1). Subsequently, the accumulated I^–^ ions promote the oxidation of the Ag contact, resulting in the formation of a AgI layer at the contact interface allowing activated Ag ions to migrate toward the bottom contact through the buffer layer.^25,41,52^ The combination of activated migrating ions and defects at a specific applied voltage (*V*_Th2_) favor the conductive filamentary formation through the buffer layers (v) abruptly switching the device to the LRS (SET2). The memristor devices, then, remain in the LRS in the reverse scan direction, necessitating a negative *V*_RESET_ (vi) to stably rupture the conductive filaments and to return back to the stabilized HRS. It is noted that the RESET process does not exhibit the two-step process. As observed in the two-step SET process, the diffusion of migrating I^–^ and V_I_^+^ (SET1) occurs prior to the formation of the Ag^+^ conductive filaments (SET2). Hence, during the reverse scan toward the negative voltages, the migrating I^–^ ions and V_I_^+^ defects already approach their relaxed state prior to the complete rupture of the conductive Ag^+^ filaments. Therefore, the single-step RESET process can be attributed to the difference in the time scales between the faster diffusion of ions and defects and the slower diffusion of the Ag ions.

In summary, we have demonstrated that a two-step resistive switching SET can be induced by the introduction of a buffer layer between the perovskite and the top contact. This additional layer turns a device that shows a volatile memory response into a nonvolatile memory. The buffer layer in conjuction with the reactive Ag contact is responsible for the abrupt SET2 process by controlling the formation of a “pool” of slow-moving ions within the buffer layer and the perovskite and by regulating the reactivity of migrating ions with Ag. Furthermore, we present a dynamical model that distinctly describes the two different switching mechamism regimes indicated by the extracted ideality factors. The experiments reveal that the current control is due to accumulated charges and interfacial reactions. Moreover, the high correlation of the model parameters suggests that the two-step SET process is governed by the same mechanism irrespective of the buffer layer. This insight into the mechanisms governing the switching response would be relevant for memristor configurations specifically tailored for targeted neural network applications with varying levels of complexity. These devices exhibit both drift and diffusive switching responses and can be utilized for adaptable implementation of versatile device designs in more diverse computational frameworks.

## Methods

The fabricated memristor device configuration consists of a fluorine-doped tin oxide (FTO)/poly(3,4-ethylenedioxythiophene) polystyrenesulfonate (PEDOT:PSS)/MAPbI_3_/buffer layer/Ag/Au structure. The two different buffer layers used are (6,6)-phenyl C61 butyric acid methyl ester (PCBM) and poly(methyl methacrylate) (PMMA). The thin buffer layers are prepared by spin coating without any additional dopants and with similar thicknesses. The fabrication method is further discussed in detail in the Supporting Information.

The film morphology of the memristor devices is inspected via cross-sectional SEM (JEOL JSM-7001F). The electrical characterizations of the memristor devices are performed inside a nitrogen-controlled glovebox in the dark using an Autolab PGSTAT204 potentiostat to minimize moisture-related degradation effects and to improve reproducibility.

The electroforming step is initiated via a voltage sweep from 0 V to 2.5 V to 0 V with a cutoff current of 50 mA, which immediately reverses the scan direction once it is reached to avoid complete device breakdown. Instead of current compliance, which limits the maximum operating current but continues the voltage scan direction, the cutoff current is implemented to observe the full switching response without a loss of information. The characteristic *I*–*V* responses of the devices are then measured via a voltage sweep from 0 V to +*V*_u_ to −*V*_l_ to 0 V, where the upper (*V*_u_) and lower (*V*_l_) voltage vertices are selected for stable, reproducible resistive switching from a HRS or OFF state to a LRS or ON state.

## Supporting Information Available

Memristor device fabrication, stabilized characteristic *I*–*V* switching response, ON state retention and endurance measurements, redox reaction of the MAPbI_3_/Ag/Au device, and dynamical model fitting resources (PDF)
Raw current-voltage data of device containing PCBM buffer layer (TXT)
Transparent Peer Review report available (PDF)
