# Supplementary material for: Mechanistic and Kinetic Analysis of Perovskite Memristors with Buffer Layers: The Case of a Two-Step Set Process
Source: J Phys Chem Lett. 2023 Feb 4;14(6):1395–402. doi: 10.1021/acs.jpclett.2c03669 (PMC9940207; doi:10.1021/acs.jpclett.2c03669)
Supplement: Supplementary file 1 — jz2c03669_si_001.pdf [file jz2c03669_si_001.pdf]

Supporting Information

Mechanistic and Kinetic Analysis of Perovskite Memristors with Buffer Layers: The Case of Two-Step Set Process

Cedric Gonzales and Antonio Guerrero*
Institute of Advanced Materials (INAM), Universitat Jaume I, 12006 Castelló, Spain.

Corresponding authors: A. Guerrero (aguerrer@uji.es)

Memristor Device Fabrication

Substrate Preparation

The fluorine-doped tin oxide (FTO) substrates were partially etched with zinc powder and 2 M HCl solution. The etched samples were individually brushed to mechanically remove the remaining residues of the etching procedure, then, were sonicated for 15 minutes each in deionized water with Hellmanex detergent solution, acetone, and isopropyl alcohol, respectively. The cleaned substrates were then dried using a nitrogen gun.

PEDOT:PSS Deposition

Prior to the deposition of the PEDOT:PSS layer, the etched and cleaned FTO substrates were subjected to a 15 min-ultraviolet ozone treatment to further remove organic contamination on the surface and improve surface wetting. The PEDOT:PSS (Heraeus Clevios P VP Al 4083) solution was filtered using a 0.45 µm PTFE syringe filter before injection onto the substrate surface. The PEDOT:PSS was, then, spin-coated on the FTO substrates at 3000 RPM with an acceleration of 1000 RPM/s for 30 s. The spin-coated samples were heated at 100 °C for 5 mins then were immediately transferred into the nitrogen-controlled glovebox.

$MAPbI_3$ Deposition

The FTO substrates with the PEDOT:PSS layer were subjected to dehydration inside the glove box at 100 °C for 10 minutes to further remove humidity during transport. . A 1.4 M $MAPbI_3$ precursor solution is prepared using PbI_2 (>98 %, TCI) and MAI (>99.99 %, Greatcell Solar) in 95 µL:1 mL dimethylsulfoxide (DMSO) (≥99.9 %, Sigma Aldrich):N,N-dimethylformamide (DMF) (99.8 %, Sigma Aldrich) solution. A 50 µL MAPBr perovskite solution was statically spin-coated onto the PEDOT:PSS layer via an anti-solvent method of 4000 RPM with 1000 RPM/s acceleration for 50 s. A 500 µL chlorobenzene (99.8 %, Sigma Aldrich) anti-solvent was injected 8 seconds after the spin coating has started. The samples were then annealed at 100 °C for 10 minutes.

Thin Undoped Buffer Layer Deposition

The buffer layers consist of a 10 mg/mL (6,6)-Phenyl C61 butyric acid methyl ester (PCBM); an electron selective layer, and a 5 mg/mL Poly(methyl methacrylate) (PMMA); an insulating polymer. The buffer layer solutions are prepared in 1 mL chlorobenze without any additional dopants. A 50 µL undoped buffer solution was dynamically spin-coated at 6000 RPM with 800 RPM/s acceleration for 30 s.

Metal Contact Deposition

Finally, a 15 nm Ag contact, followed by an 85 nm Au contact was thermally evaporated using a commercial Oerlikon Leybold Univex 250.

Stabilized Characteristic $I-V$ Switching Response

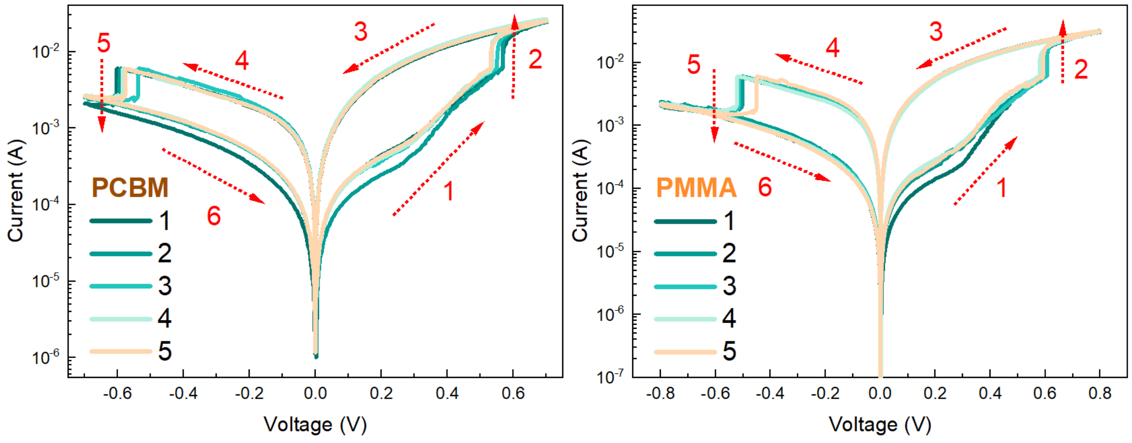

Figure S1. The representative stabilized characteristic $I-V$ switching response of the memristor devices with thin undoped (a) PCBM and (b) PMMA. The upper (V_u) and lower (V_l) voltage vertices are specifically selected for the devices to exhibit stable and reproducible resistive switching.

ON State Retention and Endurance Measurements

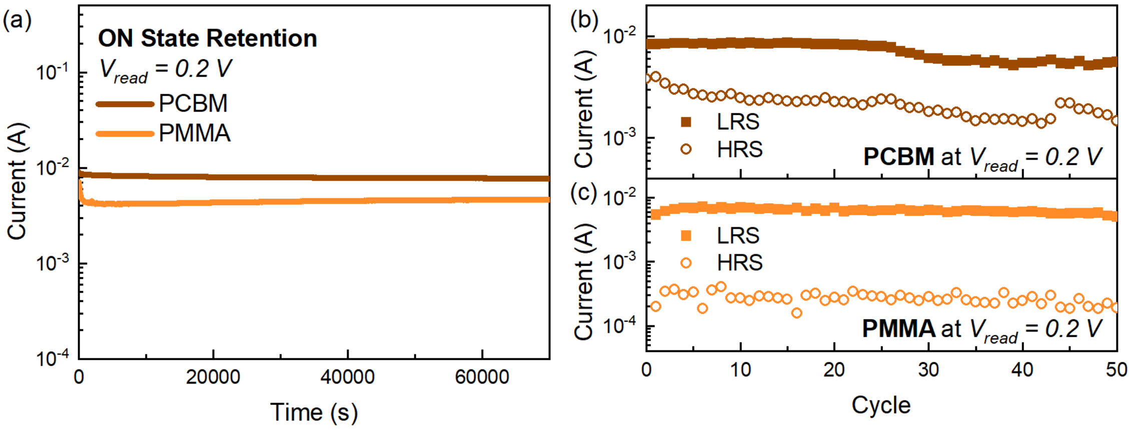

Figure S2. (a) The ON state retention times of both the PCBM and PMMA memristors by a SET voltage pulse of V_{SET} = 1.5 V for 5 s to switch the device to the ON state then subsequently measured at a read voltage of V_{read} = 0.2 V. Endurance measurements of the HRS (OFF state) and LRS (ON state) during cyclic voltammetry consecutive cycling for both the (b) PCBM and (c) PMMA devices measured at the same read voltage of V_{read} = 0.2 V.

Redox Reaction of $MAPbI_3$/Ag/Au Device

The characteristic $I-V$ response of a memristors without any thin undoped buffer layer is investigated as shown in Fig. S3. In the semi-log scale (Fig. S3a), the characteristic $I-V$ response of the device without the buffer layer ($MAPbI_3$/Ag/Au) does not exhibit a memristor resistive switching. Instead, a reduction-oxidation (redox) of Ag and I resulting to the formation and rupture of AgI at the perovskite/Ag interface is observed in the positive and negative polarities, respectively.[1-3] In the log-log scale, the $MAPbI_3$/Ag/Au

memristor device (Fig. S3b) also intially exhibits an Ohmic conduction with m_i ~ 0.93 for applied voltages below the onset of current increase of ~ 0.21 V. Moreover, the slopes of the subsequent voltage ranges (m_{ii} to m_v) calcuated via a piecewise linear fitting vary from ~0.73 up to 7.27. The low correlation of the slopes beyond the onset of current increase of the memristor without a buffer layer compared to the devices with varying buffer layers suggests that the abrupt resistive switching is intimately correlated to the filamentary formation occuring within the buffer layers.[4] Furthermore, the applied voltage range (V_{app} < 0.8 V) for memristor devices with the various thin undoped buffer layers has been reported to be insufficient to promote metal ion migration towards the perovskite bulk layer further confirming the filamentary conduction within the buffer layers.[5,6]

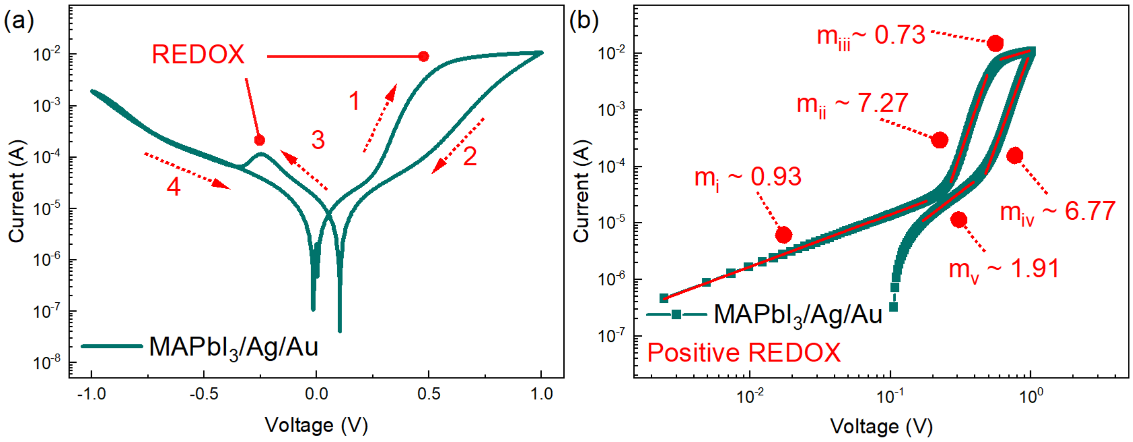

Figure S3. (a) The characteristic $I - V$ curve of the memristor device with no buffer layer in semi-log scale exhibiting a reduction-oxidation (redox) process instead of a memristive switching response with the arrows indicating the scan direction. (b) The corresponding positive scan redox in the log-log scale with the calculated slopes via piecewise linear fitting.

Dynamical Model Fitting Code

```
import numpy as np
import matplotlib.pyplot as plt
from scipy.optimize import curve_fit

# import data
pcbm = 'pcbm.csv'

with open(pcbm, 'r', encoding='utf-8-sig') as f:
    data = np.genfromtxt(f, dtype=float, delimiter=',')

# data index range
l_data = 0
u_data = 280

# fit index range
l_fit = 0
u_fit = 280

# data
us_data = data[l_data:u_data,0]
Is_data = data[l_data:u_data,1]
```

```
# fit data
us_fit = data[l_fit:u_fit,0]
Is_fit = data[l_fit:u_fit,1]

# initial guess
V_T1i = 0.7
V_m1i = 0.06
R_bi = 1e3
i_c1i = 0.05
V_T2i = 0.5
V_m2i = 0.001
i_c2i = 0.0001
c0 = [V_T1i, V_m1i, R_bi, i_c1i, V_T2i, V_m2i, i_c2i]

# upper bounds
V_T1u = np.inf
V_m1u = np.inf
R_bu = np.inf
i_c1u = np.inf
V_T2u = np.inf
V_m2u = np.inf
i_c2u = np.inf
ub = [V_T1u, V_m1u, R_bu, i_c1u, V_T2u, V_m2u, i_c2u]

# lower bounds
V_T1l = 0.0
V_m1l = 0.0
R_bl = 0.0
i_c1l = 0.0
V_T2l = 0.0
V_m2l = 0.0
i_c2l = 0.0
lb = [V_T1l, V_m1l, R_bl, i_c1l, V_T2l, V_m2l, i_c2l]

def I_app(u, V_T1, V_m1, R_b, i_c1, V_T2, V_m2, i_c2):
    f_ss = 1.0 / (1.0 + np.exp(-(u-V_T1)/V_m1))
    g_ss = 1.0 / (1.0 + np.exp(-(u-V_T2)/V_m2))
    return u/R_b + i_c1*f_ss + i_c2*g_ss

popt, pcov = curve_fit(I_app, us_fit, Is_fit, p0=c0, bounds=(lb, ub), max_nfev=10000,
ftol=1e-10)

fig, (ax1, ax2, ax3) = plt.subplots(1, 3, figsize = (15, 4))

ax1.plot(us_fit, 1000*Is_fit, 'ko', mfc = 'none', label = 'lin-lin')
ax1.plot(us_fit, 1000*I_app(us_fit, *popt), 'r-', linewidth = 3.0, label = 'fit')
ax1.set_xlabel('Voltage (V)')
ax1.set_ylabel('Current (mA)')

ax2.semilogy(us_fit, Is_fit, 'ko', mfc = 'none', label = 'log-lin')
ax2.semilogy(us_fit, I_app(us_fit, *popt), 'r-', linewidth = 3.0, label = 'fit')
ax2.set_xlabel('Voltage (V)')
ax2.set_ylabel('Current (A)')
ax2.legend(loc = 'best')
```

```
ax3.loglog(us_fit, Is_fit, 'ko', mfc = 'none', label = 'log-log')
ax3.loglog(us_fit, I_app(us_fit, *popt), 'r-', linewidth = 3.0, label = 'fit')
ax3.set_xlabel('Voltage (V)')
ax3.set_ylabel('Current (A)')
ax3.legend(loc = 'best')

fig.tight_layout()

fig, (ax1, ax2, ax3) = plt.subplots(1, 3, figsize = (15, 4))

ax1.plot(us_data, 1000*Is_data, 'ko', mfc = 'none', label = 'lin-lin')
ax1.plot(us_data, 1000*I_app(us_data, *popt), 'r-', linewidth = 3.0, label = 'fit')
ax1.set_xlabel('Voltage (V)')
ax1.set_ylabel('Current (mA)')
ax1.legend(loc = 'best')

ax2.semilogy(us_data, Is_data, 'ko', mfc = 'none', label = 'log-lin')
ax2.semilogy(us_data, I_app(us_data, *popt), 'r-', linewidth = 3.0, label = 'fit')
ax2.set_xlabel('Voltage (V)')
ax2.set_ylabel('Current (A)')
ax2.legend(loc = 'best')

ax3.loglog(us_data, Is_data, 'ko', mfc = 'none', label = 'log-log')
ax3.loglog(us_data, I_app(us_data, *popt), 'r-', linewidth = 3.0, label = 'fit')
ax3.set_xlabel('Voltage (V)')
ax3.set_ylabel('Current (A)')
ax3.legend(loc = 'best')

fig.tight_layout()
```

References

(1) Ilyas, N.; Li, C.; Wang, J.; Jiang, X.; Fu, H.; Liu, F.; Gu, D.; Jiang, Y.; Li, W. A Modified Sio 2 -Based Memristor with Reliable Switching and Multifunctional Synaptic Behaviors. *J. Phys. Chem. Lett.* **2022**, 884-893.

(2) Waser, R.; Dittmann, R.; Staikov, C.; Szot, K. Redox-Based Resistive Switching Memories Nanoionic Mechanisms, Prospects, and Challenges. *Adv. Mater.* **2009**, 21, *25-26*, 2632-2663.

(3) John, R. A.; Demirağ, Y.; Shynkarenko, Y.; Berezovska, Y.; Ohannessian, N.; Payvand, M.; Zeng, P.; Bodnarchuk, M. I.; Krumeich, F.; Kara, G.; Shorubalko, I.; Nair, M. V.; Cooke, G. A.; Lippert, T.; Indiveri, G.; Kovalenko, M. V. Reconfigurable Halide Perovskite Nanocrystal Memristors for Neuromorphic Computing. *Nat. Commun.* **2022**, 13, *1*, 1-10.

(4) Gonzales, C.; Guerrero, A.; Bisquert, J. Spectral Properties of the Dynamic State Transition in Metal Halide Perovskite-Based Memristor Exhibiting Negative Capacitance. *Appl. Phys. Lett.* **2021**, 118, *073501*.

(5) Zhu, X.; Lee, J.; Lu, W. D. Iodine Vacancy Redistribution in Organic–Inorganic Halide Perovskite Films and Resistive Switching Effects. *Adv. Mater.* **2017**, 29, *29*, 1-8.

(6) Kerner, R. A.; Zhao, L.; Harvey, S. P.; Berry, J. J.; Schwartz, J.; Rand, B. P. Low Threshold Voltages Electrochemically Drive Gold Migration in Halide Perovskite Devices. *ACS Energy Lett.* **2020**, 5, *11*, 3352-3356.
