# Supplementary material for: Mechanistic and Kinetic Analysis of Perovskite Memristors with Buffer Layers: The Case of a Two-Step Set Process
Source: J Phys Chem Lett. 2023 Feb 4;14(6):1395–402. doi: 10.1021/acs.jpclett.2c03669 (PMC9940207; doi:10.1021/acs.jpclett.2c03669)
Supplement: Supplementary file 3 — jz2c03669_si_003.pdf [file jz2c03669_si_003.pdf]

jz-2022-03669d.R1

Name: Peer Review Information for "Mechanistic and Kinetic Analysis of Perovskite Memristors With Buffer Layers: The Case of Two-Step Set Process"

First Round of Reviewer Comments

Reviewer: 1

Comments to the Author

In the manuscript Gonzales C. and Guerrero A. show a MAPbI3 perovskite memristor with two SET steps, discussing the underlying principles and models of the device operation. The major advance of this work is the comprehensive analysis of the mechanism for two-step SET perovskite memristors, before being unexplored. The authors present both qualitative description and mathematical model, well-describing ongoing processes in perovskite memristors, clearly proving the necessity of a buffer layer for two-step set operation. Such advance has immediate significance for the perovskite community as well as for researchers of emerging memory technologies for the following reasons: 1) this work shows further versatility of perovskite memristors devices; 2) this manuscript provides good insight into the devices operation principles and vital connection with migration of different ions; 3) the authors paves the path for fabrication and characterization of such two-step SET memristor devices. I consider the manuscript is well-written and scientific claims are well-supported with experimental data. Thus, I would recommend this manuscript for publication in the Journal of Physical Chemistry after addressing a few minor technical concerns, listed below.

Minor remarks

1. The authors provide a reasonable explanation of the origin of the two-step SET process. However, it isn't entirely clear, why in the case of such a device, the RESET doesn't exhibit two steps as well and whether it is possible to design a two-step RESET device. I suggest for the authors to add a bit more discussion on that matter, providing a model for the RESET part, if possible.

2. The authors show stabilized IV response in Figure 1S. It is seen that the device is rather stable for at least 5 cycles. The question is whether it is the limit of the device or whether it can sustain much more cycles? I suggest for the authors to provide more data on the stability of their device, more specifically, retention and endurance characteristics.

Reviewer: 2

Comments to the Author

The manuscript entitled "Mechanistic and Kinetic Analysis of Perovskite Memristors With Buffer Layers: The Case of Two-Step Set Process" by Gonzales, Cedric et al. reported a two-step resistive switching set

process involving a complex interplay among mobile halide ions/vacancies (I-/VI+) and silver ions (Ag+) in perovskite-based memristors with thin undoped buffer layers. Furthermore, the authors developed a dynamical model that explains the characteristic I-V curve for helping to untangle and quantify the switching regimes consistent with the experimental memristive response. Overall, this work is interesting and worthy of further study. The reviewer recommends the publication of this manuscript. The reviewer also suggests that some minor problems should to be solved.

1. Could the authors discuss more about metal halide perovskite materials for neuromorphic applications (e.g., Nature Communications 2022, 13, 7427; Matter 2022, 5, 1578; Adv. Funct. Mater. 2020, 30(46), 2005413).

2. It is difficult to obtain the detailed thickness of each layer from Figure 1(a, b). It is suggested that the author provide more detailed data support through more characterization means (e.g., AFM or HRTEM).

3. It is suggested that the author provide the original data named “pcbm” so that reviewers can verify the simulation results.

4. Some errors in References should be corrected.

Reviewer: 3

Comments to the Author

The article, “Mechanistic and kinetic analysis of perovskite memristors with buffer layers: the case of two-step set process” is well written. It reveals a phenomenon that either wasn't known before or that many authors didn't acknowledge explicitly in the materials they were working on. Authors have fabricated the device one with a PMMA buffer layer on top and another with a PDMS buffer layer, in the main article. The paper describes a two-step SET process and notes that the MAPbI3 layer without a buffer layer did not have one. The mechanism underlying this process is also mentioned and extensively detailed in the article, however, there are some aspects I would want to suggest to the authors to support the assertions made in the paper.

1. Although the authors explicitly mention the effect of the buffer layer in the log-log plot in the supplementary material, it would be great if they can show the influence of this two-step SET process in various synaptic characteristics, to show the influence of buffer layer in the plasticity of the constructed memristive device.

2. The claims using various should also be supported by impedance results.

3. What are the reasons of the low on/off ratio while there are numerous reports on high on/off ratio?

4. The last point I would like to request from the authors, is to prove the reproducibility of I-V characteristics by performing more I-V sweeps or providing the endurance-retention data in the supplementary material of this article.

5. MAPbI3-based memristors are also utilized as photonic memristor that exhibits ionization of their constituents by broadband visible light. It needs to be mentioned, under what illumination conditions, the electrical characterizations are performed for this device.

~Source: https://doi.org/10.1021/acsnano.7b07317

6. Explanation to the double layer oy electrode Ag/Au should be explicitly explained.

Author's Response to Peer Review Comments:

Dr. Antonio Guerrero
Associate Professor
Institute of Advanced Materials
Universitat Jaume I
Avda. Sos Baynat sn, 12006 Castelló, Spain
phone +34 964 387 529
email: aguerrer@uji.es

14 January 2023
Prof. Editor
Editor of the *Journal of the Physical Chemistry Letters*

Dear Prof. Editor

We thank you for considering our recent submission for publication in the *Journal of the Physical Chemistry Letters* and we are glad for the generally positive feedback from the reviewers.

We have considered all the reviewers' comments and have implemented a number of changes to improve the overall quality of the manuscript. Please, find below a point-by-point response to their comments that includes new measurements. We have also modified the manuscript to include all the formatting requirements requested by in your last communication.

We hope that after these revisions the manuscript meets the high standards of the *Journal of the Physical Chemistry Letters* and it is accepted for publication.

Best wishes,

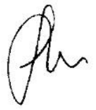

Dr Antonio Guerrero

Associate Professor
Institute of Advanced Materials
University Jaume I

Point-By-Point response to Reviewers

Reviewer: 1

Recommendation: This paper is publishable subject to minor revisions noted. Further review is not needed.

Comments:

In the manuscript Gonzales C. and Guerrero A. show a MAPbI3 perovskite memristor with two SET steps, discussing the underlying principles and models of the device operation. The major advance of this work is the comprehensive analysis of the mechanism for two-step SET perovskite memristors, before being unexplored. The authors present both qualitative description and mathematical model, well-describing ongoing processes in perovskite memristors, clearly proving the necessity of a buffer layer for two-step set operation. Such advance has immediate significance for the perovskite community as well as for researchers of emerging memory technologies for the following reasons: 1) this work shows further versatility of perovskite memristors devices; 2) this manuscript provides good insight into the devices operation principles and vital connection with migration of different ions; 3) the authors pave the path for fabrication and characterization of such two-step SET memristor devices. I consider the manuscript is well-written and scientific claims are well-supported with experimental data. Thus, I would recommend this manuscript for publication in the Journal of Physical Chemistry after addressing a few minor technical concerns, listed below.

Minor remarks:

1. The authors provide a reasonable explanation of the origin of the two-step SET process. However, it isn't entirely clear, why in the case of such a device, the RESET doesn't exhibit two steps as well and whether it is possible to design a two-step RESET device. I suggest for the authors to add a bit more discussion on that matter, providing a model for the RESET part, if possible.

   Authors' Response: We thank the reviewer for the detailed review of the manuscript. The reviewer highlights a very good point. While the first gradual activation (SET1) process is related to the drift of the migrating I^- and V_I^+ within the perovskite at lower voltages, the second abrupt activation (SET2) is related to the diffusion of Ag^+ resulting to the formation of conductive filaments at higher voltages. This suggests that the drift-related migration of ions and defects is promoted faster than the diffusion-related filamentary formation. Similarly, during the reverse scan direction towards the negative voltages, the mobile I^- and V_I^+

already begin to diffuse back to their relaxed state at low negative bias. However, the formed conductive Ag filaments only fully rupture at a higher negative bias when the ions and vacancies are already approaching their relaxed state. Therefore, the RESET process only exhibits a single step due to the difference in the time scales between the faster diffusion of ions and vacancies as compared to the slower diffusions of Ag ions eventually rupturing the conductive filaments.

We have added the following discussions in the manuscript to include the discussion of the single step RESET process to improve the discussion of the proposed switching mechanism:

"It is noted that the RESET process does not exhibit the two-step process. As observed in the two-step SET process, the drift-related migration of I^- and V_I^+ (SET1) occurs prior to the diffusion-related formation of the Ag^+ conductive filaments (SET2). Hence, during the reverse scan towards the negative voltages, the migrating I^- and V_I^+ already approach their relaxed state prior to the complete rupture of the conductive Ag^+ filaments. Therefore, the single step RESET process can be attributed to the difference in the time scales between the faster diffusion of ions and vacancies, and the slower diffusion of the Ag ions."

2. The authors show stabilized IV response in Figure 1S. It is seen that the device is rather stable for at least 5 cycles. The question is whether it is the limit of the device or whether it can sustain much more cycles? I suggest for the authors to provide more data on the stability of their device, more specifically, retention and endurance characteristics.

Authors' Response: We agree with the reviewer that the stability of the resistive switching response is important to fully describe the device performance and characteristics. As such, we have conducted the ON state retention time measurements via a set voltage of V_{SET} = 1.5 V for 5 s to switch the device to the LRS then immediately measured the ON state at a read voltage of V_{read} = 0.2 V for ~ 70,000 s. On the other hand, we have conducted the endurance measurements by cycling for more than 50 times to monitor the stability of the ON and OFF states measured at V_{read} = 0.2 V.

We have added the following discussions in the manuscript to discuss the retention times and endurance characteristics of the memristor devices:

"Both devices exhibit ON state retention times approcaching 10^5 s at a read voltage of V_{read} = 0.2 V with endurance of > 50 cycles (Supporting Information Fig. S2). It is worth noting that the memristor device configuration is designed to emphasize

the kinetics and dynamics of the resistive switching mechanism. Incorporation of large-size cation dopant, such as ethylenediammonium (en), has proved to substatially improve the device stability to record endurances of 1.2×10^4 cycles.[1]"

We have also added the ON state rentention and endurance data in the Supporting Information for reference to the additional characterizations:

ON State Retention and Endurance Measurements

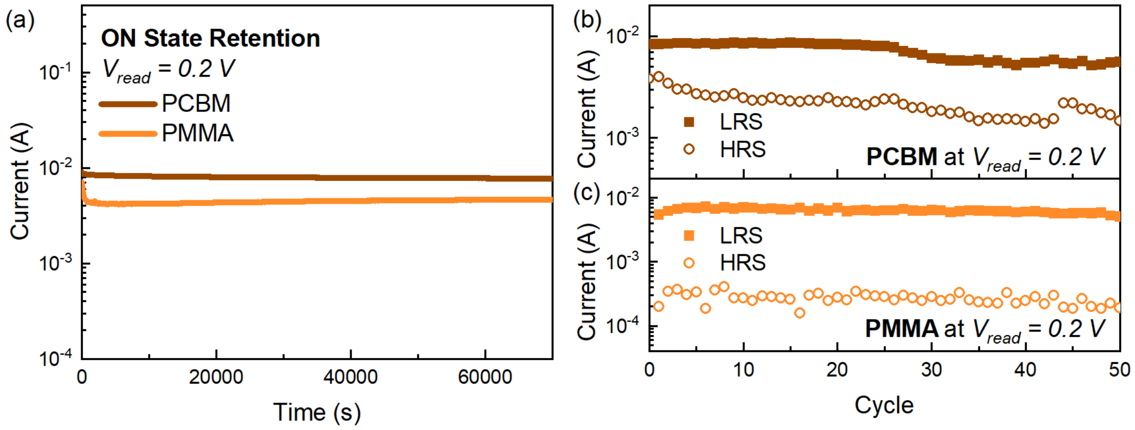

Figure S2. (a) The ON state retention times of both the PCBM and PMMA memristors by a SET voltage pulse of V_{SET} = 1.5 V for 5 s to switch the device to the ON state then subsequently measured at a read voltage of V_{read} = 0.2 V. Endurance measurements of the HRS (OFF state) and LRS (ON state) during cyclic voltammetry consecutive cycling for both the (b) PCBM and (c) PMMA devices measured at the same read voltage of V_{read} = 0.2 V.

Reviewer: 2

Recommendation: This paper is publishable subject to minor revisions noted. Further review is not needed.

Comments:

The manuscript entitled "Mechanistic and Kinetic Analysis of Perovskite Memristors with Buffer Layers: The Case of Two-Step Set Process" by Gonzales, Cedric et al. reported a two-step resistive switching set process involving a complex interplay among mobile halide ions/vacancies (I-/VI+) and silver ions (Ag+) in perovskite-based memristors with thin undoped buffer layers. Furthermore, the authors developed a dynamical model that explains the characteristic I-V curve for helping to untangle and quantify the switching regimes consistent with the experimental memristive response. Overall, this work is interesting and worthy of further study. The reviewer recommends the publication of this manuscript. The reviewer also suggests that some minor problems should to be solved.

1. Could the authors discuss more about metal halide perovskite materials for neuromorphic applications (e.g., Nature Communications 2022, 13, 7427; Matter 2022, 5, 1578; Adv. Funct. Mater. 2020, 30(46), 2005413).

   Authors' Response: We appreciate the review for the detailed review of our work. We added the suggested references and others in relation to their applications to further highlight the advances in metal halide perovskite-based memristors for neuromorphic applications:

   "Perovskite-based memristive devices have been demostrated to function as artificial synapses exhibiting essential synaptic behaviors for neuromuscular systems, pupil reflex, and for light-sensitive optogenetic applications.[2-5]"

2. It is difficult to obtain the detailed thickness of each layer from Figure 1(a, b). It is suggested that the author provide more detailed data support through more characterization means (e.g., AFM or HRTEM).

   Authors' Response: We appreciate the reviewer for pointing this out. We appreciate the reviewer for pointing this out as he/she is totally right in fact the thickness could still be lower than that we reported in the previous version of the manuscript. A buffer layer with a thickness of 20-30 nm should clearly be visible in the cross-section SEM. The fact that we do not see it clearly in the SEM suggest that it is in the limit of our system resolution which is in the order of 5-10 nm. We

have modified this figure in the current version of the manuscript. It should be noted that the use of AFM to measure thickness requires the ultrathin film to end with a step on top of a highly flat surface wherein the step height between the substrate and the film surface can be measured.[6] However, as the $MAPbI_3$ films crystallize with grain boundaries, the surface roughness measured via AFM can range from 8 – 12 nm. As this surface roughness is of the same order of magnitude as the PCBM and PMMA thin film thicknesses, accurate measurement using AFM is very challenging.[7,8] On the other hand, unfortunately, we currently do not have an adequate HRTEM system to be able for high resolution thickness measurement of the films.

3. It is suggested that the author provide the original data named “pcbm” so that reviewers can verify the simulation results.

   Authors’ Response: The raw data of the PCBM memristor $I-V$ response (“pcbm.csv”) is attached.

4. Some errors in References should be corrected.

   Authors’ Response: All references in both the main manuscript and the Supporting Information have been updated to follow the Journal of Physical Chemistry Letters format.

Reviewer: 3

Recommendation: This paper is publishable subject to minor revisions noted. Further review is not needed.

Comments:

The article, "Mechanistic and kinetic analysis of perovskite memristors with buffer layers: the case of two-step set process" is well written. It reveals a phenomenon that either wasn't known before or that many authors didn't acknowledge explicitly in the materials they were working on. Authors have fabricated the device one with a PMMA buffer layer on top and another with a PDMS buffer layer, in the main article. The paper describes a two-step SET process and notes that the MAPbI3 layer without a buffer layer did not have one. The mechanism underlying this process is also mentioned and extensively detailed in the article, however, there are some aspects I would want to suggest to the authors to support the assertions made in the paper.

1. Although the authors explicitly mention the effect of the buffer layer in the log-log plot in the supplementary material, it would be great if they can show the influence of this two-step SET process in various synaptic characteristics, to show the influence of buffer layer in the plasticity of the constructed memristive device.

   Authors' Response: We thank the reviewer for the detailed review and positive comments of our work. We agree with the reviewer that the demonstration of the influence of two-step process in various synaptic characteristics would further emphasize the importance of the switching mechanisms for specific applications. However, we emphasize that this is a mechanistic study and we are highlighting the observed two-step SET process and its kinetics. Nevertheless, we will soon be working on the demonstration of the various synaptic functions of our memristive devices with a more complete experimental design.

2. The claims using various should also be supported by impedance results.

   Authors' Response: We acknowledge that having the impedance response would further complement the electrical characterization of the devices. We have previously demonstrated the impedance spectral evolution of the dynamic state transitions of perovskite-based memristors with and without an interfacial buffer layer.[9] The full investigation and analysis of the impedance spectral evolution of the effect of different buffer layer configurations and the perovskite formulations deserve their own studies but are beyond the scope of this work.

3. What are the reasons of the low on/off ratio while there are numerous reports on high on/off ratio?

   Authors' Response: We thank the reviewer for this clarification. Explaining the exact reason on the variations in the reported ON/OFF ratios is not straightforward. We note that the memristive response is measured by the total current vs. voltage. In this case, the measured current levels are highly dependent on the device's effective size. The effective device sizes of the reports with higher ON/OFF ratios are in the ~100 μm^2 – 5 mm^2 scales. In these scales, the HRS current levels are significantly lower than in our devices, which have ~0.25 cm^2 in size. With our relatively larger device areas, we observe the average conductance making it more susceptible for perovskite film defects that increase the current levels of the OFF state. Moreover, depending on the switching mechanism, the LRS could reach the same current levels as our devices in the case of conductive filamentary formation resulting to high ON/OFF ratios. Therefore, the difference in the device effective size plays a major role in the difference in ON/OFF ratios of our devices compared to the other reported devices. However, the devices' effective size is just one of several factors that can affect the characteristics of memristors as further discussed by Sakhatsyi et.al. in the supplementary information of the article.[1]

4. The last point I would like to request from the authors, is to prove the reproducibility of I-V characteristics by performing more I-V sweeps or providing the endurance-retention data in the supplementary material of this article.

   Authors' Response: We agree with the reviewer that the stability of the resistive switching response is important to fully describe the device performance and characteristics. As such, we have conducted the ON state retention time measurements via a set voltage of V_{SET} = 1.5 V for 5 s to switch the device to the LRS then immediately measured the ON state at a read voltage of V_{read} = 0.2 V for ~ 70,000 s. On the other hand, we have conducted the endurance measurements by cycling for more than 50 times to monitor the stability of the ON and OFF states measured at V_{read} = 0.2 V.

   We have added the following discussions in the manuscript to discuss the retention times and endurance characteristics of the memristor devices:

   "Both devices exhibit ON state retention times approcaching 10^5 s at a read voltage of V_{read} = 0.2 V with endurance of > 50 cycles (Supporting Information Fig. S2). It is worth noting that the memristor device configuration is designed to emphasize the kinetics and dynamics of the resistive switching mechanism. Incorporation of large-size cation dopant, such as ethylenediammonium (en), has proved to

substatially improve the device stability to record endurances of 1.2×10^4 cycles.[1]"

We have also added the ON state rentention and endurance data in the Supporting Information for reference to the additional characterizations:

ON State Retention and Endurance Measurements

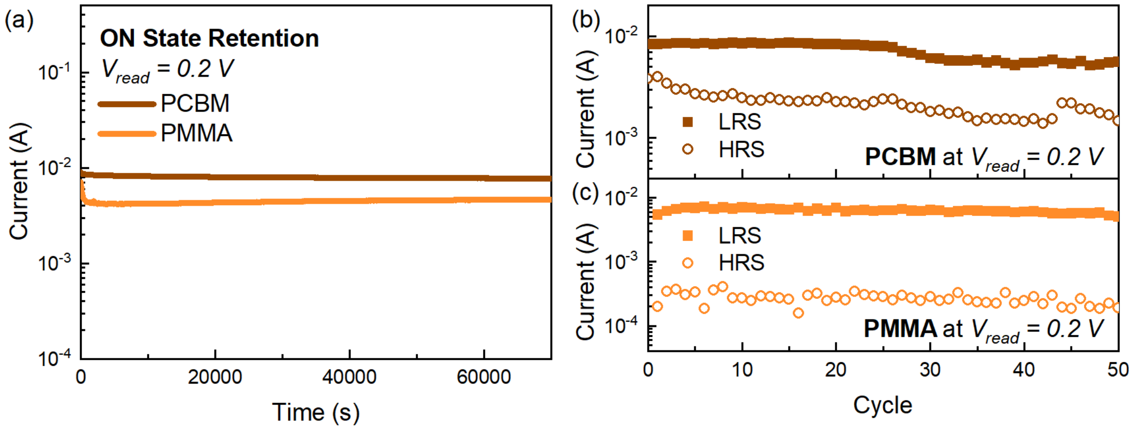

Figure S1. (a) The ON state retention times of both the PCBM and PMMA memristors by a SET voltage pulse of V_{SET} = 1.5 V for 5 s to switch the device to the ON state then subsequently measured at a read voltage of V_{read} = 0.2 V. The endurance measurements of the HRS (OFF state) and LRS (ON state) for both the (b) PCBM and (c) PMMA devices measured at the same read voltage of V_{read} = 0.2 V.

5. MAPbI3-based memristors are also utilized as photonic memristor that exhibits ionization of their constituents by broadband visible light. It needs to be mentioned, under what illumination conditions, the electrical characterizations are performed for this device. Source: https://doi.org/10.1021/acsnano.7b07317

   Authors' Response: We have added the following discussions in the manuscript to further highlight the recent advancements in $MAPbI_3$-based memristor applications:

   "Perovskite-based memristive devices have been demostrated to function as artificial synapses exhibiting essential synaptic behaviors for neuromuscular systems, pupil reflex, and for light-sensitive optogenetic applications.[2-5]"

6. Explanation to the double layer of electrode Ag/Au should be explicitly explained.

   Authors' Response: We appreciate the reviewer for pointing this out. We have added the following discussions in the manuscript to highlight the rationale of the Ag/Au contact.

"In addition, the thin Ag/Au contact is used to control and modulate the interactivity of the mobile I^+ ions with Ag to prevent the formation of a excessively thick AgI structure with low ionic conductivity.[10]"

References

(1) Sakhatskyi, K.; John, R. A.; Guerrero, A.; Tsarev, S.; Sabisch, S.; Das, T.; Matt, G. J.; Yakunin, S.; Cherniukh, I.; Kotyrba, M.; Berezovska, Y.; Bodnarchuk, M. I.; Chakraborty, S.; Bisquert, J.; Kovalenko, M. V. Assessing the Drawbacks and Benefits of Ion Migration in Lead Halide Perovskites. *ACS Energy Lett.* **2022**, 3401-3414.

(2) Gong, J.; Wei, H.; Liu, J.; Sun, L.; Xu, Z.; Huang, H.; Xu, W. An Artificial Visual Nerve for Mimicking Pupil Reflex. *Matter* **2022**, 5, *5*, 1578-1589.

(3) Gong, J.; Yu, H.; Zhou, X.; Wei, H.; Ma, M.; Han, H.; Zhang, S.; Ni, Y.; Li, Y.; Xu, W. Lateral Artificial Synapses on Hybrid Perovskite Platelets with Modulated Neuroplasticity. *Adv. Funct. Mater.* **2020**, 30, *46*, 1-10.

(4) Liu, J.; Gong, J.; Wei, H.; Li, Y.; Wu, H.; Jiang, C.; Li, Y.; Xu, W. A Bioinspired Flexible Neuromuscular System Based Thermal-Annealing-Free Perovskite with Passivation. *Nat. Commun.* **2022**, 13, *1*, 1-11.

(5) Zhu, X.; Lu, W. D. Optogenetics-Inspired Tunable Synaptic Functions in Memristors. *ACS Nano* **2018**, 12, *2*, 1242-1249.

(6) Gesang, T.; Fanter, D.; Höper, R.; Possart, W.; Hennemann, O. D. Comparative Film Thickness Determination by Atomic Force Microscopy and Ellipsometry for Ultrathin Polymer Films. *Surf. Interface Anal.* **1995**, 23, *12*, 797-808.

(7) Ma, J.-Y.; Ding, J.; Yan, H.-j.; Wang, D.; Hu, J.-s. Temperature-Dependent Local Electrical Properties of Organic−Inorganic Halide Perovskites: In Situ Kpfm and C-Afm Investigation. *ACS Appl. Mater. Inter.* **2019**, 11, 21627-22163.

(8) Niu, Q.; Huang, W.; Tong, J.; Lv, H.; Deng, Y.; Ma, Y.; Zhao, Z. Understanding the Mechanism of Pedot: Pss Modification Via Solvent on the Morphology of Perovskite Films for Efficient Solar Cells. *Synthetic Met.* **2018**, 243, 17-24.

(9) Gonzales, C.; Guerrero, A.; Bisquert, J. Spectral Properties of the Dynamic State Transition in Metal Halide Perovskite-Based Memristor Exhibiting Negative Capacitance. *Appl. Phys. Lett.* **2021**, 118, *073501*.

(10) Teymourinia, H.; Gonzales, C.; Gallardo, J. J.; Salavati-Niasari, M.; Bisquert, J.; Navas, J.; Guerrero, A. Interfacial Passivation of Perovskite Solar Cells by Reactive Ion Scavengers. *ACS Applied Energy Materials* **2021**, 4, *2*, 1078-1084.
